# The therapeutic effect of bromocriptine in combination with spironolactone in patients with primary aldosteronism: a hypothesis generating pilot study

**DOI:** 10.18632/oncotarget.20670

**Published:** 2017-09-06

**Authors:** Vin-Cent Wu, Che-Hsiung Wu, Ya-Wen Yang, Kuo-How Huang, Chia-Hui Chang, Shao-Yu Yang, Yen-Hung Lin, Kwan-Dun Wu, The NRPB Kidney Consortium

**Affiliations:** ^1^ Division of Nephrology, National Taiwan University Hospital, Taipei, Taiwan; ^2^ Division of Nephrology, Taipei Tzu Chi Hospital, Buddhist Tzu Chi Medical Foundation, Taipei, Taiwan; ^3^ School of Medicine, Tzu Chi University, Hualien, Taiwan; ^4^ Division of General Surgery, Department of Surgery, National Taiwan University Hospital, Taipei, Taiwan; ^5^ Department of Internal Medicine, Urology, National Taiwan University Hospital, Taipei, Taiwan; ^6^ NRPB, National Research Program for Biopharmaceuticals, Taipei, Taiwan

**Keywords:** primary aldosteronism, bromocriptine, spironolactone

## Abstract

**Background:**

Dopamine D2-like receptors are attenuated in aldosterone producing adenoma, lead to overproduction of aldosterone in affected patients, and thus reported to serve as a potential treatment target for primary aldosteronism. The D2 dopamine receptor agonist bromocriptine has been used clinically for reducing tumor mass of pituitary adenomas of lactotroph origin. The aim of the present study was to assess the efficacy of adding bromocriptine to spironolactone in the biochemical control of primary aldosteronism.

**Methods:**

Thirty patients (15 aldosterone producing adenoma) received bromocriptine treatment with dose titration to a daily dose of 7.5mg. Urine aldosterone and potassium excretion ratio of all patients were compared based on the result of metoclopramide test at baseline.

**Results:**

On the basis of response to metoclopramide at baseline, the proportions of patients with lower urine aldosterone and urine potassium level after taking bromocriptine for six months were higher in the high metoclopramide response group. Initial aldosterone-renin ratio and high metoclopramide response at baseline were independent predictors of a decrease in aldosterone secretion after a six–month course of bromocriptine. The effects of bromocriptine added to spironolactone to reduce aldosterone secretion and potassium excretion in primary aldosteronism dissipated at 9 month after the initial treatment.

**Conclusions:**

In this pilot study, we found that short-term addition of bromocriptine to spironolactone improved the biochemical control of primary aldosteronism. Dopamine agonist is more effective in patients with high baseline aldosterone-renin ratio and those sensitive to metoclopramide stimulation. However, this effect dissipated after 9 months.

**Clinical trial registry information:**

ClinicalTrials. Gov number: NCT00451672; https://www.clinicaltrial.gov/ct2/show/NCT00451672?term=NCT00451672&rank =1; trial registry name: The Therapeutic Effect of Bromocriptin in Patients With Primary Aldosteronism.

## INTRODUCTION

Primary aldosteronism (PA), which is characterized by inappropriate production of aldosterone, affects 5-13% of patients with hypertension [1]. Aldosterone antagonists (e.g.spironolactone) usually bring about satisfactory blood pressure (BP) control in patients with bilateral adrenal hyperplasia (BAH) [2], although medication must be taken for life. Aldosterone-producing adenoma (APA) is the most common subtype that is curable by adrenalectomy [3], but, not all patients respond to surgery [4] and patients with bilateral adenoma would not benefit from unilateral adrenalectomy [5].

Aldosterone secretion is regulated by an inhibitory dopaminergic mechanism [6, 7]. Pharmacological and autoradiographic studies have identified D2-like receptors in the adrenal zona glomerulosa [8]. Numbers of D2-like receptors are reduced in APA, leading to overproduction of aldosterone, and thus serve as a potential target of treatment for PA [7].

In several *in vivo* and human studies, administration of dopaminergic antagonists, such as metoclopramide (MCP) has been shown to cause a rise in plasma aldosterone levels [9], but not plasma cortisol levels [8]. We have previously reported that PA patients with higher number of D2 receptors showed greater aldosterone secretion in response to a MCP test [7, 8]. In APA patients the increase in aldosterone level following administration of MCP was inversely related to the level of aldosterone synthase mRNA (CYP11B2) in the tumors.

The D2 receptor agonist bromocriptine (BMC) has been used clinically to reduce the mass of pituitary adenomas of lactotroph origin [10]. D2 receptors are pleiotropic receptors in that activating them inhibits adenylyl cyclase, resulting in inhibition of voltage-gated calcium currents and in activation of potassium conductance [11]. Early studies suggested that anterior pituitary hyperplasia was correlated with hypersecretion of prolactin [12] and the use of D2 receptor agonists resulted in a reduction in tumor mass. Our previous study demonstrated that BMC inhibited angiotensin II-stimulated cell proliferation in primary cultures of normal human adrenal cortex and APA. Whether dopamine receptor agonists can be used in the clinical management of PA remains to be clarified. We therefore carried out a prospective study to examine the additive effect of BMC treatment as a supplement to spironolactone by looking at spontaneous aldosterone secretion in PA over a 24-hour period. Since the therapeutic effect of spironolactone on PA is well-established, we did not expect the addition of BMC to add significantly to the biochemical control of PA. We thought that the suppressive effect of BMC on aldosterone secretion might only be detectable in selected patients. Patients with increased D2 receptor expression among adrenal gland, who exhibited high aldosterone levels after MCP test, were more likely to have hyperaldosterone that was regulated by the dopaminergic system. Therefore, we hypothesis that MCP test response could serve as a predictor for therapeutic effect of BMC. Our aim was to identify independent predictors of enhancement of aldosterone suppression by BMC in this hypothesis generating pilot study. Patients with high and low aldosterone levels after administration of MCP were compared. We aimed to assess initial response to MCP and then the therapeutic effect of BMC; therefore we did not include a placebo control group in our design.

## RESULTS

### Sample

**Figure 1:**
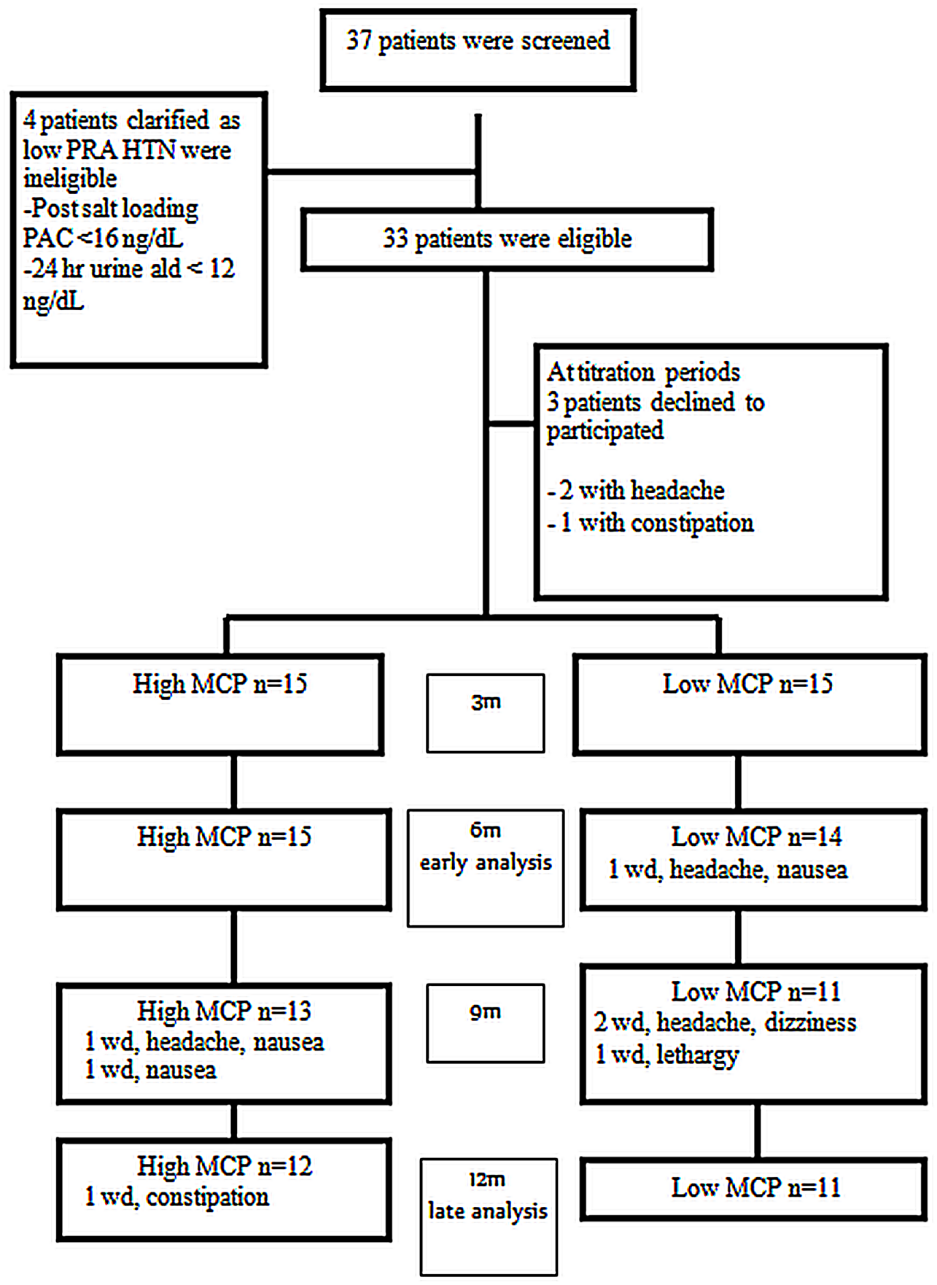
Flow chart for patient enrollment HTN, hypertension; MCP, metoclopramide; PAC, plasma aldosterone concentration; PRA, plasma renin activity; wd, withdraw.

Among 37 enrolled patients with ARR> 35 during the study period and signed informed consent, 33 patients (18 women and 15 men; mean age 51.0 ± 11.4 years) had been diagnosed with PA. Three patients withdrew during the first month while BMC dose was being titrated (Figure 1). The final cohort of 30 patients was divided into high (n = 15) and low MCP (n = 15) response group based on the results of the MCP test.

There were 15 patients with APA. Seven patients withdrew from BMC treatment during the course of study. The most common side effect was headache (4 patients) followed by nausea (3 patients). One patient withdrew because of excessive lethargy, and one patient experienced constipation (Figure 1).

### Primary outcomes: time-dependent variables after taking BMC

**Figure 2:**
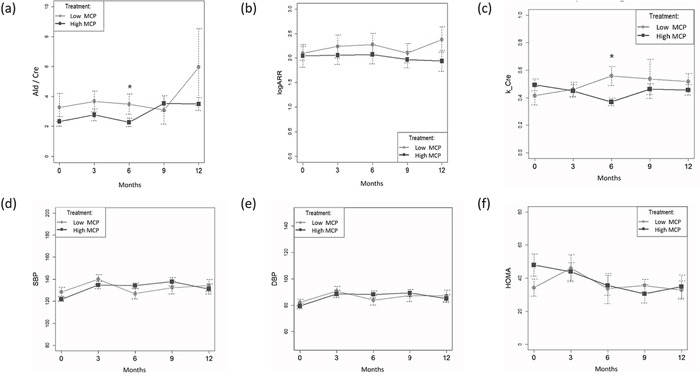
**(a)** Daily urine aldosterone/creatinine **(b)** Log ARR **(c)** urine potassium/creatinine **(d)** Systolic Blood Pressure (SBP) **(e)** Diastolic Blood Pressure (DBP) **(f)** HOMA-IR (mean± SD) at different time points for patients with high and low MCP response at baseline by generalized estimating equations (GEE) method were expressed* p< 0.05 at time point compared with baseline result; all values are reported as the mean ± SD. (a) The Ald/cre between group, p = 0.341. Changes in time period contributed to changes in Ald/cre after 6 months (GEE, standardized regression coefficient for time (-0.871)[95% CI,(-1.614)–(-0.128)] in both modalities, p = 0.021 for first-order interaction). (b) The logARR between groups, p = 0.340. (c) The K/Cre between group, p = 0.758. Changes in time period contributed to changes in K/Cre after 6 months (GEE, standardized regression coefficient for time (-0.088)[95% CI,(-0.154)–(-0.021)] between groups, p = 0.0098 for first-order interaction). (d) The SBP between group, p = 0.554. (e) The DBP between groups, p = 0.712. (f) The HOMA between group, p = 0.424.

Serum ARR, SBP, DBP and HOMA-IR did not significantly change during the study period. Six months after starting BMC treatment the urine aldosterone/creatinine ratios were 3.472 ± 2.261 and 2.268 ± 1.073 in the low and high MCP response groups respectively. The GEE model indicated that change in urine aldosterone level and potassium excretion over the six-month treatment period were different significantly in the high and low MCP response groups (p = 0.021 and p = 0.0098, respectively) (Figure 2). The categories of antihypertensive medications decreased from 1.13 ± 0.82 to 0.90 ± 0.86 with no statistical significance (p>0.05).

### Secondary analysis: independent predictors of better suppression of aldosterone secretion after a six-month course of BMC

The accumulated plasma aldosterone concentration (PAC) after MCP stimulation, calculated by area under the curve correlated inversely with serum potassium level (p= 0.004, r= -0.523)

**Figure 3:**
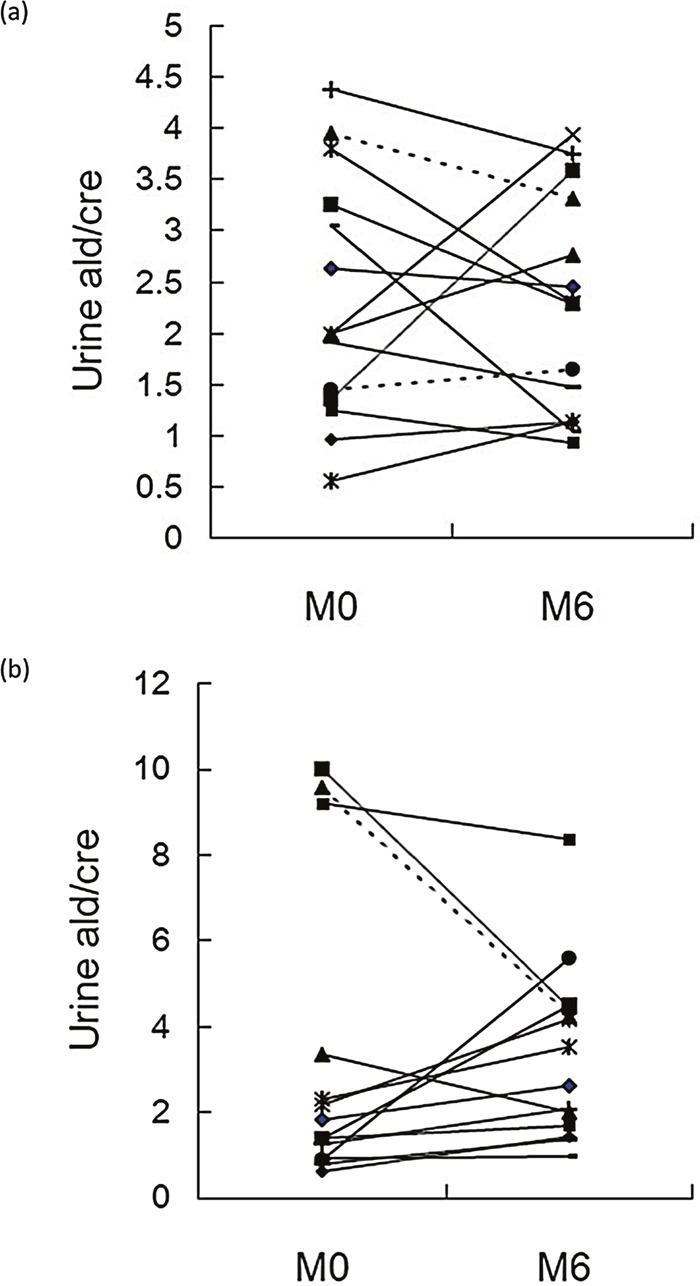
Change in aldosterone excretion over 6 months of BMC treatment: M0 (baseline) to M6 **(a)** high MCP group; **(b)** low MCP group (p= 0.004). BMC, bromocriptine, MCP, metoclopramide; urine ald/ cre (aldosterone / creatinine), (ng/dL / mg/dL)

Patients were divided into high and low MCP response group on the basis of response to MCP at baseline. At enrolment the two MCP response groups were similar with respect to demographic variables, aldosterone profile and metabolic profile (Table 1). The proportions of patients with lower urine aldosterone and potassium levels after taking BMC for six months were higher in the high MCP response group ((p= 0.004 and p =0.011 respectively) (Figure 3). However, these differences were not significant when patients had been taking BMC for 9 and 12 months. There were no time dependent serial changes between low and high MCP response groups by ARR, SBP, DBP and HOMA-IR (Figure 2).

**Table 1:** Clinical and biochemical characteristics of study patients as per treatment

	Low MCP	High MCP	*p*
Patients (n)	15	15	NA
**Demography**			
Sex (M)	8 (53.3)	5 (33.3)	0.462
Age (y/o)	51.7 ± 10.5	47.8 ± 10.0	0.307
Charlson comorbidity index ^¶^	0.4 ± 0.6	0.2 ±0.4	0.314
MAP (mmHg)	117.9 ± 13.4	114.1 ± 8.3	0.372
Latency of HTN (yr)	10.8 ± 8.2	8.9 ± 6.2	0.464
BMI (kg/m2)	26.4 ± 4.0	25.5 ± 3.3	0.521
**Aldosteronism profile**			
APA	8(53.3)	7 (46.7)	0.999
Potassium (mmole/L)	3.6± 0.8	3.7 ± 0.4	0.577
PRA (ng/mL/hr)	0.48 ± 0.37	0.96 ± 1.05	0.107
PAC (ng/dL) ^#^	49.2(43.3-62.7)	45.2(33.5-67.7)	0.767
Log [ARR]	2.1 ± 0.6	2.0 ± 0.9	0.851
Urine K /creatinine	0.42 ± 0.26	0.49 ± 0.15	0.351
**Metabolic profile**			
Cholesterol (mg/dl)	195.1±22.0	206.1± 50.2	0.443
Triglyceride (mg/dl)	158.7 ± 79.9	193.9 ± 173.6	0.482
HDL (mg/dl)	45.1± 10.8	42.1 ± 6.6	0.359
Uric Acid	6.7 ± 1.4	6.6 ± 1.7	0.824
FPG (mg/dL)	92.1 ± 12.0	100.7 ± 13.7	0.084
HOMA-IR (mU/L·mmol/L)	34.0 ± 20.5	47.9 ± 26.1	0.124
**Cardiac function profile**			
Left ventricular mass	209.9 ± 64.7	183.3 ± 51.1	0.236
LVEF	65.23 ± 7.3	67.8 ± 5.1	0.285
**Categories of hypertensive drugs before recruitment**			
α- blockers	3 (20.0)	2 (13.3)	0.999
β- blockers	3 (20.0)	2 (13.3)	0.999
Calcium channel blockers	12 (80.0)	7 (46.2)	0.128
ACEI/ ARB	1 (6.7)	4 (26.7)	0.330
**Early outcome at 6 month**			
Urine ald / creatinine*	3.472 ± 2.261	2.268 ± 1.073	0.091
Urine K / creatinine*	0.558 ± 0.22	0.370 ± 0.01	0.011
Decreased ald secretion*	1 (7.1)	9 (64.3)	0.004
**Late outcome at 12 month**			
urine ald / creatinine**	5.952 ± 7.727	3.489± 1.501	0.291
urine K / creatinine**	0.517 ± 0.198	0.456 ± 0.138	0.386
Decreased ald secretion**	1 (11.1)	1(8.3)	0.999
Tumor size	1.0 ± 0.3	1.0 ± 0.2	0.785

ACEI, angiotensin-converting enzyme inhibitors; ald, aldosterone; APA, aldosterone producing adenoma; ARB, angiotensin II receptor blockers; ARR, aldosterone-renin ratio (ng/dL per ng/mL/h); BMI, body mass index; FPG, fasting plasma glucose; HDL, high-density lipoprotein; HOMA-IR, homeostasis model assessment for insulin resistance; HTN, hypertension; K, potassium; MAP, mean arterial blood pressure; LVEF, left ventricular ejection fraction; PAC, plasma aldosterone concentration; PRA, plasma renin activity.

Patients were withdrawn from antihypertensive medications at least 21 days before the study, with the exception of calcium antagonist or alpha-blockers.

Data were provided as the mean values ± standard deviation, Significance was determined by Wilcoxon signed rank test in nonparametric distribution.

^¶^ Charlson Comorbidity Index contains 19 categories of comorbidity and predicts the ten-year mortality for a patient who may have a range of co-morbid conditions.

^#^ Median (interquartile range)

* with one patient withdrawal before 6 month in low MCP response group

** with eligible 11 patients in low MCP and 12 patients in high MCP groups

To convert potassium in mmol/L to mEq/L, multiple by 1; BUN in mg/dL to mmol/L, multiple by 0.375; eGFR in mL/min to mL/s, multiple by 0.01667; PAC in ng/dL to nmol/L, multiple by 0.02774; PRA in ng/mL/hr to ng/(Lxs), multiple by 0.2778.

Patients who had increased aldosterone excretion had higher serum ARR at enrollment (2.45 ± 0.97 vs. 1.89 ± 0.49, p= 0.097), and more of them were in the high MCP response group (90% vs. 31.6 %, p=0.004) (Table 2).

**Table 2:** Clinical parameters at enrollment related to primary outcome of intention to treatment

	Increased aldosterone secretion	Decreased aldosterone secretion	*p*
Patients (n)	19	10	
**Demography**			
Sex (M)	10 (52.6)	2 (20.0)	0.126
Age (y/o)	49.7± 11.0	49.1 ± 9.5	0.862
Charlson comorbidity index	0.37 ± 0.6	0.1 ± 0.32	0.125
MAP (mmHg)	115.1 ± 11.7	116.2 ± 10.0	0.798
Latency of HTN (yr)	9.7± 8.1	10.1 ± 6.0	0.885
BMI (kg/m2)	26.5 ± 3.9	24.5 ± 2.8	0.176
**Aldosteronism profile**			
APA	11 (57.9)	4 (40)	0.450
Potassium (mmole/L)	3.7 ± 0.7	3.6± 0.5	0.553
PRA (ng/mL/hr)	0.76 ± 0.74	0.67 ± 1.00	0.809
PAC (ng/dL) ^#^	44.0(38.9-55.2)	56.9(42.7-82.1)	0.347
Log [ARR]	1.89 ± 0.49	2.45± 0.97	0.097
Urine ald /creatinine	2.36 ± 2.65	3.57 ± 2.50	0.247
Urine K /creatinine	0.41± 0.23	0.52± 0.17	0.178
**Metabolic profile**			
Cholesterol (mg/dl)	205.2± 45.3	195.6 ± 20.8	0.442
Triglyceride (mg/dl)	183.3 ± 135.1	164 ± 145.2	0.732
HDL (mg/dl)	44.1± 10.2	42.8 ± 6.8	0.809
Uric Acid	6.8 ± 1.3	6.47 ± 1.9	0.673
FPG (mg/dL)	93.56 ± 12.0	102.9 ± 15.5	0.149
HOMA-IR (mU/L·mmol/L)	36.5 ± 20.7	45.3 ± 27.0	0.387
**Categories of hypertensive drugs before recruitment**			
α- blockers	2 (20.0)	3 (15.8)	0.999
β- blockers	2 (20.0)	2 (10.5)	0.592
Calcium channel blockers	6 (60.0)	12(63.2)	0.999
ACEI/ ARB	3 (30.0)	2 (10.5)	0.306
High MCP	6 (31.6)	9 (90)	0.004
Tumor size	1.0 ± 0.3(11 APA patients)	1.2 ± 0.1(4 APA patients)	0.370

ACEI, angiotensin-converting enzyme inhibitors; ald, aldosterone; APA, aldosterone producing adenoma; ARB, angiotensin II receptor blockers; ARR, aldosterone-renin ratio (ng/dL per ng/mL/h); BMI, body mass index; FPG, fasting plasma glucose; HDL, high-density lipoprotein; HOMA-IR, homeostasis model assessment for insulin resistance; HTN, hypertension; K, potassium; MAP, mean arterial blood pressure; MCP, metoclopramide; PAC, plasma aldosterone concentration; PRA, plasma renin activity;

Patients were withdrawn from antihypertensive medications at least 21 days before the study, with the exception of calcium antagonist or alpha-blockers.

Data were provided as the mean values ± standard deviation, Significance was determined by Wilcoxon signed rank test in nonparametric distribution.

^#^ Median (interquartile range)

The body- mass index is the weight in kilograms divided by the square of the height in meters

To convert potassium in mmol/L to mEq/L, multiple by 1; BUN in mg/dL to mmol/L, multiple by 0.375; eGFR in mL/min to mL/s, multiple by 0.01667; PAC in ng/dL to nmol/L, multiple by 0.02774; PRA in ng/mL/hr to ng/(Lxs), multiple by 0.2778.

Multivariate regression models including demographic variables, aldosterone profile, metabolic profile and categories of hypertensive drug (Table 3) indicated that initial ARR (OR = 12.9, 95% CI: 1.003- 166.7, p = 0.050) and high MCP response at baseline (OR = 125, 95% CI: 2.639- 1000, p = 0.014) were independent predictors of a decrease in aldosterone secretion after a six- month course of BMC.

**Table 3:** Relative Risks and 95% Confidence Intervals of Independent Factors to predict decreased aldosterone secretion by Multivariate Logistic Regression

Variable	β	Wald	p	Odds ratio	95% CI	*p* value (adjusted)
Log ARR at enrollment	2.559	3.850	0.049	12.9	1.003 - 166.7	0.050
High MCP group	4.768	6.055	0.014	125	2.639 - 1000	0.014

ARR, aldosterone rennin ratio; MCP, metoclopramide.

### Tumor size

In the ten APA patients who completed the entire course of BMC, tumor size was unchanged regardless of whether aldosterone secretion increase or decrease (1.02 ± 0.24 cm to 1.07 ± 0.28 cm (p= 0.064) in five increased aldosterone secretion group and 1.03 ± 0.28 cm to 1.07 ± 0.34 (p=0.344) in five decreased aldosterone secretion group).

## DISCUSSION

This is the first clinical study to document the suppressive effect of a D2 receptor agonist on aldosterone excretion in PA. We assessed the effectiveness of a combined therapy including a D2 receptor agonist in clinical practice and found that adding a D2 receptor agonist to spironolactone improved the biochemical control of aldosterone secretion. Adjunct treatment with D2 receptor agonist could, therefore, be considered for PA patients with poor biochemical control. Although our study is limited by the small sample size and side-effects of BMC, our data highlight the clinical importance of diagnosing aldosteronism and suggested a method of managing bilateral lesions. Furthermore, our study also provided evidence of the potential benefits of this kind of combination therapy in routine clinical practice, as it provides a demonstration of the short-term effectiveness of combined therapy outside the tight confines of clinical trials.

### Complication

The utility of BMC appears to be limited owing to the disappointingly high rates of subjective complications and the serious side-effects that affect5-10% of prolactinoma patients taking high does [20]. The appearance of side-effects (nausea, dizziness and postural hypotension) is a limiting factor for continued treatment. In this trial, nausea was the common limiting adverse event. In the majority of patients nausea occurred during initial titration of the drug. Despite the poor efficacy of the first dopamine receptor agonists, agents such as cabergoline appear promising [21]. An earlier review concluded that cabergoline should be the preferred dopamine agonist for medical management of giant prolactinomas owing to its excellent efficacy and tolerability [22]. Though cabergoline may be better tolerated than BMC, burning, crawling, itching, numbness, prickling, ‘pins and needles’ and tingling sensation are still problems and, importantly, it is also far more expensive than BMC. For these reasons we chose to use BMC in this study, rather than newer agents such as cabergoline.

### Bromocriptine resistance

At 6 month, nearly 34.5% of aldosteronism patients responded to BMC treatment, in terms of aldosterone and potassium excretion. In patients with prolactinoma treated with dopamine agonists, prolactin levels failed to normalize in about 25 % of patients treated with BMC.

There are a number of mechanisms that may account for resistance to dopamine agonists in patients with prolactinoma and many may also apply to aldosteronism. These mechanisms include (1) reduced medication absorption, resulting in lower circulating levels and lower concentrations of medication reaching the tumor [23]; (2) lower numbers of D2 receptors on resistant tumors [24]; (3) reduction in the affinity of D2 receptors for the dopamine agonists [25]; (4) altered signal transduction in the resistant tumor once the dopamine agonist binds to the D2 receptor [26]. Possible treatment strategies for patients with prolactinoma that is resistant to dopamine agonists include changing to another dopamine agonist or increasing the dose of the dopamine agonist as long as there is continued response to the dose increases and no adverse effects with higher doses (25). Similar treatment strategies could be applied to BMC-resistant aldosteronism patients.

### Bromocriptine-responsive aldosteronism

Patients with increased D2 receptor density, who exhibited high aldosterone levels after MCP test, were more likely to have aldosteronism that was regulated by the dopaminergic system [8]. After 6 months of treatment with BMC, patients with a high MCP response showed a better response in terms of the surrogate markers of urine aldosterone and potassium excretion than patients with low MCP response. Interestingly, patients with high ARR showed a good response to BMC treatment. Although aldosterone secretion in patients with APA is autonomous, there is evidence that aldosterone secretion is subject to dopaminergic inhibition [27]. APA patients may have lower D2 receptor expression than other PA patients and healthy controls. However, a recent study by Rossitto et al. showed that acute D2 dopaminergic receptor blockade during adrenal vein sampling exerted a prominent secretagogue effect on aldosterone in APA patients [28]. The results of our study suggest that it is reasonable to hypothesize that patients with high ARR and a stronger response to MCP will benefit from treatment with D2 receptor agonists. This finding suggests a new therapeutic target for PA.

Physiologically, aldosterone secretion occurs in a pulsatile manner throughout the day rather than at a steady rate [29]. Measuring aldosterone in a 24-h urine sample has the advantage of avoiding circadian variations in plasma levels [30, 31]. Therefore, 24-h urinary aldosterone level (Uald-24 h) measurement should be a better indicator in terms of disease severity in clinical practice than plasma aldosterone measurement. However, 24-h urine collections are time consuming and cumbersome. Our earlier study showed that if the effect of differences in the rates of creatinine excretion is accounted for, then the aldosterone-to-creatinine ratio in random urine samples offers comparable diagnostic accuracy to that of Uald-24 h in PA patients [19].

### Metabolic change

A considerable body of evidence from animal and clinical studies suggests that reduced dopaminergic neurotransmission is involved in the pathogenesis of metabolic syndrome [32]. D2 receptor activation has beneficial effects on body composition and fuel metabolism in obese individuals with metabolic syndrome [33]. Insulin resistance, measured in terms of HOMA-IR, was lower after 12 months of BMC treatment and there was a downwards trend in insulin resistance throughout the treatment period. BMC treatment can reduce body fat stores, lipolysis and plasma levels of free fatty acids and triglycerides. It can also promote protein turnover, lean body mass accretion and insulin-stimulated glucose disposal [34]. In line with our findings, clinical studies have shown that treatment with D2 receptor agonists improves the metabolic profile of obese non-diabetic and diabetic individuals [32]. BMC can improve metabolic syndrome, as indicated by HOMA-IR, in acromegalic patients [35]. Our pilot study demonstrated that short-term addition of BMC to spironolactone can improve the metabolic profile of PA patients and decrease their aldosterone secretion. However, BMC was the first dopamine agonist with relatively higher treatment resistance rate and relative low efficacy. Further research using other dopamine agonists is needed to evaluate the therapeutic effects of dopamine agonist treatment for patients with PA.

### Limitations

Our study has some limitations that should be acknowledged. First, although patients in the high MCP response group were more likely to show reduced urinary level of aldosterone and urine potassium after 6 months of BMC treatment than those in the low MCP response group, BMC did not improve their blood pressure. Although the biochemical data was relevant, many factors will relate to blood pressure change especially with high salt intake. Patients in our study followed an ad lib diet and for ethical reasons adjustments to antihypertensive medication were permitted during the study period. This might account for the biochemistry-phenotype discrepancy we observed. Second, nearly one third of patients withdrew from the study due to the side-effects of BMC, which may confound the study results. We analyzed the result with intention-to-treat method and compared the primary outcome at 6 months after treatment in order to minimize inconsistencies. Finally, the MCP test results correlated with urine K / creatinine ratio and decreased ald secretion at 6 months after treatment. However, urine ald / creatinine level at 6 month was with a borderline significance with the MCP test results (p= 0.09). This partial inconsistency may be due to the limited patient sample sizes that resulted in underpowered statistical analyses to provide conclusive results. Further large-sample randomized controlled trial is needed to confirm this finding.

## MATERIALS AND METHODS

### Ethics statement

The study complied with the Declaration of Helsinki and was approved by National Taiwan University Hospital Research Ethics Committee (IRB No. 9461700402). The methods were carried out in accordance with the approved guidelines. All participants received comprehensive written information and signed a consent form before inclusion into the study.

### Study protocol

**Figure 4:**
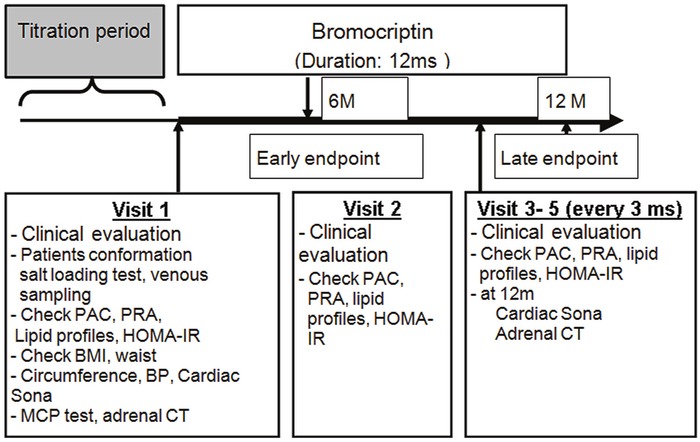
Study protocol for clinical parameter examinations BP, blood pressure; Sona, sonography; BMI, body mass index; CT, computer tomography; MCP, metoclopramide; HOMA-IR, homeostasis model assessment for insulin resistance; IHA; idiopathic hyperaldosteronism; PAC; plasma aldosterone concentration; PRA, plasma renin activity.

We conducted a prospective trial among PA patients to determine whether adding BMC to a spironolactone treatment regimen would decrease aldosterone secretion 6 months later (early endpoint) and 12 months later (late endpoint) (Figure 4). Aldosterone excretion was evaluated by dividing urine aldosterone level (ng/dL) by urine creatinine level (mg/dL) and, potassium excretion was evaluated by dividing urine potassium level (mmol/L) by urine creatinine level (mg/dL). Prior to enrollment all patients were taking 50 mg spironolactone. During the first month of the study, patients were seen face-to-face once a week, after this they were seen every 3 months until the end of the study or until early termination. BP measurements, biochemical data and aldosterone profiles were obtained every 3 months from enrollment. Patients were contacted 30 days after stopping MCP to record any adverse events that occurred after cessation. Body mass index (BMI) was calculated in kilograms per meter-squared (m2). Insulin resistance expressed as homeostasis model assessment index of insulin resistance (HOMA-IR) was calculated as fasting glucose (mmol/liter) x fasting insulin (mIU/liter) /22.5][13]. Comorbidities at enrollment were recorded and scored according to the Charlson comorbidity index [14], a weighted index that takes into account the number and seriousness of underlying disorders.

The primary aim was to evaluate temporal changes in aldosterone profile, metabolic factors and BP. The secondary aim was to identify factors that predicted improved aldosterone secretion at 6 months. BP was measured by a trained research assistant with a mercury sphygmomanometer with brachial cuff adjusted to the patient's brachial circumference. BP was measured in the right arm after the patient had been seated for 5 min. Three measurements were taken at 5-minute intervals and the average was used in analyses. A decrease in aldosterone excretion was defined as a decrease of at least 10% in urine aldosterone /creatinine ratio attenuated. The timeline of the study protocol is outlined in Figure 4.

### Eligibility

The eligibility criteria were as follows: age between 18 and 75 years of age; BMI <35 kg/m2; HbA1C level ≤7%; confirmed diagnosis of PA according to standard protocol (supplementary file) between July 1, 2007 and June 30, 2009. Patients with acute disease, depression or, a recent large change in body weight (> 5 kg in the previous 3 months) and patients who were pregnancy or using oral contraceptives were excluded. Additional exclusion criteria were current, chronic (greater than 10 days) use of prescription sympathomimetic drugs, ergot alkaloid derivatives or abortive migraine medications and any clinically significant comorbid condition, e.g. uncontrolled hypertension, New York Heart Classification (NYHC) III-IV congestive heart failure, chronic kidney disease (stage 3 or worse), cancer in the previous 5 years.

### Metoclopramide test

All subjects received Metoclopramide (MCP) test at initial screening.[8]. Briefly, all antihypertensive medications were discontinued for at least 21 days before the study. Calcium channel antagonist (diltiazem) and/or alpha-blocker (doxazosin) were administered as needed to control systolic blood pressure below 140 mmHg. Medications that might interfere with the renin-aldosterone axis, such as steroids, sex hormones, licorice, and non-steroidal anti-inflammatory drugs, were also withheld during the study period. At 9:00am, patients were asked to remain recumbent for an hour. A baseline blood sample was taken at 10:00am and then a bolus of 20 mg MCP was administered intravenously. Another blood sample was taken immediately after MCP administration and four further samples were at 10, 20, 30, and 60 min after the MCP infusion. Equal numbers of patients were assigned to the high and low MCP response groups on the basis of the receiver-operating characteristic curve analysis.

BMC (Parlodel 2.5mg) dosage was titrated to a maximum dose of 7.5 mg/d over the four-week period after enrollment. The pharmacy's Investigational Drug Service coordinated the study drug preparation. The antihypertensive drugs regimen (including spironolactone) was not changed after enrollment. Dosage of antihypertensive drugs was adjusted every 3 months on the basis of BP to maintain systolic BP below 140 mmHg. Patients returned for assessments of BP, hemogram and biochemical data every 3 months (Figure 4). After enrollment symptoms such as nausea and constipation and total amount of antihypertensive drugs were recorded systematically.

### Diagnostic criteria

The diagnosis of APA was established in hypertensive patients with elevated aldosterone-renin ratio (ARR), TAIPAI score more than 60% and evidence for lateralized disease by adrenal CT, NP59 scintigraphy or AVS. Idiopathic hyperaldosteronism (IHA) was classified in patients without evidence for lateralized disease as previously report (Test 1 and Supplementary Figure 1).

### Imaging studies

A computerized tomographic (CT) scan of the adrenal glands with a non-ionic iodinated contrast agent was performed in all patients with PA, with at least 3 mm contiguous slices in the presence of a normal surround. Although there were no strict measurements of normal adrenal size, the CT scan was considered abnormal when an area thicker than 10 mm was detected. All tumor diameters were measured, and the maximal tumor diameter (expressed in millimeters) was considered in all adenomas for further analysis.

### Assays

Aldosterone concentration was measured by a commercial radioimmunoassay kit (Aldosterone Maia Kit, Adaltis Italia S.p.A., Bologna, Italy). The lowest detectable concentration of aldosterone is 10.0 pg/mL. The normal range of aldosterone is 70-350 pg/mL in the upright position. Plasma renin activity (PRA) was measured as the generation of angiotensin I *in vitro* using a commercially available radioimmunoassay kit (DiaSorin, Stillwater, MN, USA). Its normal range is 2.63±1.32 ng/mL/h in the upright position. The mean (standard deviation [SD]) intra and interassay coefficients of variation (CVs) for the PRA assay were 1.9 (5.0%) and 4.5 (5.2%), respectively.

### Statistical analysis

The data were presented as the mean values ± standard deviation. To explore the factors for early decreased aldosterone secretion, a logistic regression analysis was used to determine all the above factors. The Mann-Whitney U test was used to determine the difference between the two groups, and the Wilcoxon signed rank test was used for paired analysis within the same group [13].

To compare the temporal course of responses of the two MCP response groups to BMC in terms of variables such as aldosterone, potassium excretion, systolic blood pressure (SBP), diastolic blood pressure (DBP) and HOMA, we fitted marginal linear regression models using the generalized estimating equations (GEE) method [15, 16]. We calculated standardized regression coefficients and their 95% confidence intervals (CIs). GEE is an effective way of achieving higher power with smaller samples or fewer repeated measurements in both complete datasets and datasets with missing values [17, 18]. Statistical analyses were performed with SPSS for Windows, version 15.0 (SPSS Inc., Chicago, IL, USA) and SAS software, Version 9.1.3 (SAS Institute Inc., Cary, NC, USA).

Skewed variables aldosterone and aldosterone-to-renin ratio (ARR) were transformed to achieve normal distributions. The significance level was set at p≦ 0.05.

### Power of the study

Based on data from previously reported study, we assumed the mean value ± standard deviation of urine aldosterone/ creatinine ratio of PA patients to be 3.17 ± 2.32 ng/mg creatinine [19]. The probability is 75 % that the study will detect the additive effect of BMC treatment on spironolactone at a two-sided 0.05 significance level, if the true difference of urine aldosterone/ creatinine ratio between high and low MCP response groups is 2 ng/mg creatinine. A study size of 30 patients would be needed to enter into this two-treatment study performed with PASS software (version 2008, NCSS, Kaysville, Utah)

### Registry of the study

The study has been registered on ClinicalTrials.gov with the registration number: NCT00451672. Because of a change of the principal investigator, there was a delay in registering this study (after enrolment of participants started). The authors confirm that all ongoing and related trials for this intervention are registered.

## CONCLUSION

In this pilot study we showed that in the short term, addition of BMC to spironolactone can reduce aldosterone secretion. Patients with high baseline response to MCP and high ARR will benefit from taking BMC as an adjunct to spironolactone treatment, although the effect dissipates after 9 months.

## SUPPLEMENTARY MATERIALS AND FIGURES



## Abbreviations

APA: aldosterone-producing adenoma
ARR: aldosterone-to-renin ratio
BAH: bilateral adrenal hyperplasia
BMC: bromocriptine
BMI: body mass index
D2R: dopamine D2 receptor
DBP: diastolic blood pressure
GEE: generalized estimating equations
HOMA-IR: homeostasis model assessment index of insulin resistance
IHA: idiopathic hyperaldosteronism
MCP: metoclopramide
PA: primary aldosteronism
PAC: plasma aldosterone concentration
PRA: plasma renin activity
SBP: systolic blood pressure
