# Supplementary material for: The therapeutic effect of bromocriptine in combination with spironolactone in patients with primary aldosteronism: a hypothesis generating pilot study
Source: Oncotarget. 2017 Sep 6;8(44):77609–21. doi: 10.18632/oncotarget.20670 (PMC5652803; doi:10.18632/oncotarget.20670)
Supplement: Supplementary file 1 [file oncotarget-08-77609-s001.pdf]

The therapeutic effect of bromocriptine in combination with spironolactone in patients with primary aldosteronism: a hypothesis generating pilot study

SUPPLEMENTARY MATERIALS

Patients were enrolled from the following hospitals

This study included two medical centers (National Taiwan University Hospital (NTUH), Taipei, Taiwan; Taipei University Hospital, Taipei, Taiwan) and five regional hospitals (Cardinal Tien Hospital, New Taipei City, Taiwan; Taipei Tzu Chi Hospital, New Taipei City, Taiwan; Yun- Lin Branch of NTUH, Douliou City, Taiwan; Hsin-Chu Branch of NTUH, Hsin-Chu City, Taiwan; Zhongxing Branch of Taipei City Hospital, Taipei, Taiwan).

Our standard protocol to identify primary aldosteronism (PA) and functional laterization

The diagnosis of primary aldosteronism was established in hypertensive patients on the basis of the following criteria [1–4](Supplementary Figure 1):

Confirmation

Fulfillment of the following three conditions confirmed a diagnosis of PA

(1) autonomous excess aldosterone production evidenced with an aldosterone-renin ratio (ARR) > 35; (2) a TAIPAI score larger than 60% [5]; (3) post-saline loading PAC > 10 ng/dL, or PAC/PRA > 35 (ng/dL)/(ng/mL/h) shown in a post-capotopril/losartan test, or PAC > 6 ng/dL indicated by a fludrocortisone suppression test [4]. (Abbreviations: PAC, plasma aldosterone concentration; PRA, plasma renin activity).

Lateralization

(1) APA was identified on the basis on the following four criteria: (1) autonomous excess aldosterone production evidenced with an ARR > 35, a TAIPAI score larger than 60% [5], and post-saline loading PAC > 10 ng/dL; (2) adenoma evidenced with a CT scan for pre-operative evaluation [6]; (3) lateralization of aldosterone secretion at adrenal vein sampling (AVS) or during dexamethasone suppression NP-59 SPECT/CT [6]; (4) pathologically proven adenoma after an adrenalectomy for those who underwent operations, and subsequent emergence of either a cure pattern of hypertension without anti-hypertensive agents or improvement in hypertension with biochemical cure [3, 4, 7].

(2) Idiopathic hyperaldosteronism (IHA) was distinguished on the basis of the following four criteria: (1) autonomous excess aldosterone production evidenced with an ARR > 35, a TAIPAI score larger than 60% [5], and post-saline loading PAC > 10 ng/dL; (2) evidence of bilateral diffuse enlargement indicated by a CT scan for pre-operative evaluation; (3) non-lateralization of aldosterone secretion at AVS or during dexamethasone suppression NP-59 SPECT/CT [6]; (4) evidence of diffuse cell hyperplasia reported in follow-up pathology studies for those with operations.

REFERENCES

1. Sechi LA, Novello M, Lapenna R, Baroselli S, Nadalini E, Colussi GL, Catena C. Long-term renal outcomes in patients with primary aldosteronism. JAMA. 2006; 295: 2638-2645.
2. Wu VC, Chang HW, Liu KL, Lin YH, Chueh SC, Lin WC, Ho YL, Huang JW, Chiang CK, Yang SY, Chen YM, Wang SM, Huang KH, et al. Primary aldosteronism: diagnostic accuracy of the losartan and captopril tests. Am J Hypertens. 2009; 22: 821-827.
3. Kuo CC, Wu VC, Huang KH, Wang SM, Chang CC, Lu CC, Yang WS, Tsai CW, Lai CF, Lee TY, Lin WC, Wu MS, Lin YH, et al. Verification and evaluation of aldosteronism demographics in the Taiwan Primary Aldosteronism Investigation Group (TAIPAI Group). J Renin Angiotensin Aldosterone Syst. 2011; 12: 348-357.
4. Chao CT, Wu VC, Kuo CC, Lin YH, Chang CC, Chueh SJ, Wu KD, Pimenta E, Stowasser M. Diagnosis and management of primary aldosteronism: an updated review. Ann Med; 45: 375-383.
5. Wu VC, Yang SY, Lin JW, Cheng BW, Kuo CC, Tsai CT, Chu TS, Huang KH, Wang SM, Lin YH, Chiang CK, Chang HW, Lin CY, et al. Kidney impairment in primary aldosteronism. Clin Chim Acta. 2011; 412: 1319-1325.
6. Yen RF, Wu VC, Liu KL, Cheng MF, Wu YW, Chueh SC, Lin WC, Wu KD, Tzen KY, Lu CC; TAIPAI Study Group. 131I-6beta-iodomethyl-19-norcholesterol SPECT/CT for primary aldosteronism patients with inconclusive adrenal venous sampling and CT results. J Nucl Med. 2009; 50: 1631-1637.
7. Wu VC, Chao CT, Kuo CC, Lin YH, Chueh SJ, Wu KD. Diagnosis and management of primary aldosteronism. Acta Nephrologica. 2012; 26: 111-120.

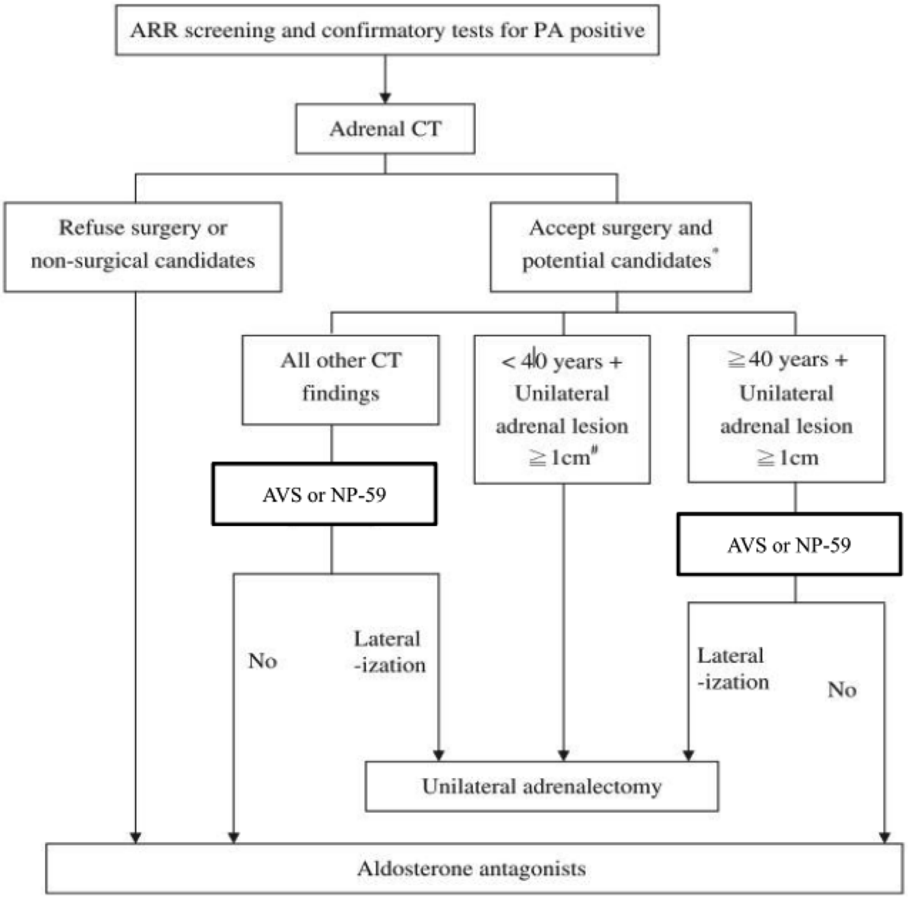

Supplementary Figure 1: The subtype-differentiating protocol of the TAIPAI group [3, 4, 7].
* Abbreviations: AVS, adrenal venous sampling; APA, aldosterone-producing adenomas; ARR, aldosterone-to-renin ratio; CT, computed tomography, IHA, idiopathic hyperaldosteronism; NP-59- SPECT, I-131-6-beta-iodomethyl-19-norcholesterol single-photon emission computed tomography.
